# Long-term follow-up of patients with relapsed/refractory multiple myeloma after BCMA CAR-T-cell therapy

**DOI:** 10.3389/fimmu.2025.1650568

**Published:** 2025-09-12

**Authors:** Yuanyuan Hao, Zhen Wang, Lei Zhang, Yanliang Bai, Xiaoli Yuan, Jing Yang, Li Jiang, Junwei Niu, Wei Cheng, Wei Li, Zhoufeng Huang, Yuqing Chen, Kai Sun, Zunmin Zhu

**Affiliations:** ^1^ Department of Hematology, Institute of Hematology, Henan Key Laboratory of Stem Cell Differentiation and Modification, Henan Provincial People’s Hospital, Zhengzhou University People’s Hospital, Zhengzhou, China; ^2^ Institute of Hematology, Henan Engineering Research Center of CAR-T-Cell Therapy and Transformation, Henan Provincial People’s Hospital, Zhengzhou University People’s Hospital, Zhengzhou, China

**Keywords:** multiple myeloma, cellular immunotherapy, BCMA CAR-T, long-term follow- up, CAR-T-cell therapy

## Abstract

**Background:**

B-cell maturation antigen (BCMA)-directed chimeric antigen receptor (CAR) T-cell therapy has demonstrated potent short-term efficacy in patients with relapsed/refractory multiple myeloma (R/R MM); however, long-term clinical data remain limited. Here, we report extended follow-up outcomes from our single-center experience.

**Methods:**

Between August 20, 2018, and December 31, 2021, 11 patients with R/R MM received BCMA-targeted CAR-T-cell therapy at our center. Preconditioning consisted of cyclophosphamide and fludarabine chemotherapy, followed by infusion of 1–5×10^6^ CAR^+^ T cells/kg. We evaluated overall response rate (ORR), long-term efficacy, safety profiles, and their correlations with clinical/disease characteristics.

**Results:**

The ORR was 72.7% (8/11), including 6 complete remissions (54.5%) and 2 partial/very good partial remissions. With a median follow-up of 23 months (range: 2–63 months), 75% (6/8) of the responders remained relapse-free, and 4 patients (50%) were alive at the time of data cutoff. The median progression-free survival (PFS) and overall survival (OS) of responders both reached 35 months. In terms of safety, most patients experienced moderate cytokine release syndrome (CRS), with 2 cases of grade 3–4 CRS.

**Conclusion:**

BCMA CAR-T-cell therapy exhibits favorable safety and efficacy in advanced R/R MM. Long-term follow-up confirmed durable responses in 50% of the advanced R/R MM patients who responded to the treatment (4/8).

## Introduction

Multiple myeloma (MM), the second most common hematologic malignancy worldwide, is characterized by clonal bone marrow plasma cells and classic “CRAB” manifestations: hypercalcemia, renal insufficiency, anemia, and lytic bone lesions (1). The introduction of novel therapeutic strategies—including immunomodulatory drugs (IMiDs), proteasome inhibitors (PIs), monoclonal antibodies, and selective nuclear export inhibitors—has significantly improved patient outcomes. Nevertheless, most patients ultimately develop relapsed or refractory disease and encounter increasingly limited treatment options (2). Patients with relapsed/refractory MM (R/R MM) have a poor prognosis, with a median overall survival (OS) of only 8–12.4 months, which highlights the critical need for more effective therapeutic strategies (3, 4).

The emergence of chimeric antigen receptor (CAR) T-cell therapy that targets B-cell maturation antigen (BCMA) has revolutionized the treatment landscape for R/R MM, particularly in patients with limited therapeutic options (5). This approach has demonstrated remarkable efficacy, with reported overall response rate (ORR) ranging from 80% to 100% across clinical trials and real-world evidence (6, 7). Despite the remarkable short-term efficacy of BCMA CAR-T-cell therapy, approximately 50% of patients experience relapse or disease progression during long-term follow-up (8, 9). Multiple factors impact progression-free survival (PFS) and OS, including disease characteristics (e.g., tumor burden, extramedullary disease, and depth of response) and T-cell characteristics (e.g., CAR-T-cell expansion, T-cell subtype composition, and T-cell exhaustion) (8, 10). However, long-term clinical data concerning the efficacy of BCMA-based CAR-T cells and a detailed characterization of infused CAR-T cells remain insufficient.

This single-center study comprehensively assessed the long-term efficacy and safety of BCMA CAR-T-cell therapy in 11 patients with R/R MM. We further investigated the kinetics of CAR-T cells and B-cell and immunoglobulin G levels, which may be associated with durable remission, following CAR-T-cell infusion.

## Methods

### Patients

Between August 20, 2018 and December 31, 2021, 11 patients with R/R MM were administered BCMA-targeted CAR-T-cell therapy in our department. This study included two clinical trials (ChiCTR1900023624 and NCT03975907) conducted during distinct periods, both of which utilized BCMA-targeted CAR-T-cell products. Among the enrolled patients, only patient 8 was part of the NCT03975907 trial, while all the other patients were included in the ChiCTR1900023624 trial. All patients met the International Myeloma Working Group diagnostic criteria for R/R MM (11). All patients provided written informed consent, and all procedures adhered to the principles of the Declaration of Helsinki and its subsequent amendments.

### CAR-T-cell production

In the ChiCTR1900023624 clinical trial, BCMA CAR-T cells were generated from autologous T cells collected via leukapheresis. A lentiviral vector was used to construct a second-generation BCMA-targeted CAR, which consists of 4-1BB costimulatory domains and CD3ζ signaling domains. CAR-T cells were generated in our laboratory according to the procedure discussed below. Peripheral blood mononuclear cells (PBMCs) were collected via leukapheresis, after which CD3^+^ T cells were isolated using anti-CD3/anti-CD28 Dynabeads (Cat. no. 40203D; Thermo Fisher Scientific, USA). Activated CD3^+^ T cells were transduced after 24–48 hours with lentivirus encoding the CAR construct (anti-BCMA scFv/4-1BB/CD3ζ) at an MOI of 5–10. Cells were cultured in X-Vivo 15 medium (Lonza Group, Ltd., Basel, Switzerland) supplemented with 300 U/ml IL-2. On days 12–14, flow cytometry (FCM) was used to analyze the BCMA CAR-T-cell transduction efficiency, after which quality control was performed prior to cell infusion. In the NCT03975907 clinical trial, a CAR-T-cell product (Zevor-cel, CT053) was generated through the transduction of T cells with a lentivirus encoding a CAR. This CAR construct included a fully human BCMA-specific single-chain variable fragment (scFv, 25C2), a human CD8α hinge domain, a CD8α transmembrane domain, a 4-1BB costimulatory domain, and a CD3ζ activation domain (12). The CAR-T cells were manufactured by CARSGEN Therapeutics and transported to our department in liquid nitrogen for storage and subsequent use.

### CAR-T-cell treatment

For the 10 patients in the ChiCTR1900023624 trial, the lymphodepletion chemotherapy regimen comprised cyclophosphamide at a dosage of 500 mg/m^2^ per day for 2 days and fludarabine at 30 mg/m^2^ per day for 4 days. On day 0, patients received an intravenous infusion of BCMA CAR-T cells at a dosage of 1–5×10^6^ cells/kg. Notably, this clinical trial was initially designed to evaluate the safety and efficacy of sequential BCMA-CAR-T and CD138-CAR-T-cell therapy for R/R MM. However, in the first two enrolled patients, our attempts to generate CD138-CAR-T cells were unsuccessful, as we were unable to detect CD138-CAR-T cells via FCM prior to infusion. Consequently, these two patients did not receive CD138-CAR-T-cell infusions, and we did not proceed with manufacturing CD138-CAR-T cells for subsequent patients. For Patient 8 in the NCT03975907 trial, the lymphodepletion chemotherapy regimen consisted of fludarabine at 25 mg/m^2^ and cyclophosphamide at 300 mg/m^2^ administered daily for 3 consecutive days. The patient received the recommended dose of 1.5 × 10^8^ BCMA CAR-T cells on day 0. Post-infusion, all patients were closely monitored with routine laboratory tests and proactive management to prevent and address CAR-T-cell–associated toxicity.

### Long-term assessments

The response to CAR-T-cell therapy was assessed according to the International Myeloma Working Group consensus criteria (13). Long-term follow-up was conducted from the date of CAR-T-cell infusion until the cutoff date or patient death. Information regarding CAR-T-cell infusion and subsequent therapies was retrieved from the medical records of our department. Other treatment-related information from the local hospital was obtained via phone interviews and records. The ORR was defined as the proportion of patients who achieved a partial response (PR) or better. OS was calculated from the date of CAR-T-cell infusion to the date of death or the cutoff date. PFS was defined as the time from CAR-T-cell infusion to disease progression or death from any cause. Minimal residual disease (MRD) was evaluated by FCM of bone marrow aspirates, and the kinetics of CAR-T cells in peripheral blood were also monitored using FCM.

### Adverse events

Adverse events were graded in accordance with the National Cancer Institute Common Terminology Criteria for Adverse Events (CTCAE) version 5.0. Cytokine release syndrome (CRS) was graded according to the criteria proposed by Lee et al. (14). Cytokines, including IL-2, IL-4, IL-6, TNF-α, and IFN-γ, were assessed by FCM using a cytokine detection kit (RAISECARE, China).

### Statistical analysis

Descriptive statistical analyses involved calculating the medians along with the minimum and maximum values for continuous variables and determining the counts and percentages for categorical variables. The probabilities of PFS and OS were estimated using the Kaplan–Meier method. All the statistical analyses were performed using GraphPad Prism 9.

## Results

### Patients

Between August 20, 2018, and October 31, 2021, 11 patients with R/R MM received BCMA-targeted CAR-T-cell therapy in our department (**Table 1**). The median patient age was 58 years (range: 54–70 years), and 6 were male (54.5%). Five patients (45.4%) had the light chain subtype, four (36.4%) had the IgG subtype, and two (18.2%) had the IgD subtype. Six patients (54.5%) were classified as International Staging System (ISS) stage II, and four (36.4%) were classified as stage III. Extramedullary disease (EMD) was detected in 2 patients (18.2%). The median number of prior treatment lines was 4 (range: 2–9), and 2 patients (18.2%) received autologous stem cell transplantation (ASCT) before CAR-T-cell infusion. The median BCMA expression in MM cells was 66.23% (range: 3.17%–98.02%), and the median serum β2-microglobulin concentration was 2.7 mg/L (range: 1.17–6.19 mg/L). The baseline characteristics of the patients are summarized in **Table 2**.

**Table 1:** Clinical characteristics of patients.

No.	Age	Sex	Monoclonal globulin	ISS stage	Previous therapy lines	Prior ASCT	Extramedullary disease	BCMA on MM cells	Infusion dose of BCMA CAR-T	Transduction efficiency
1	57	M	Light chain κ	I	3	No	No	63.58%	1.34×10^8^	21.14%
2	58	M	Light chain λ	II	4	No	EM-E	66.23%	3.14×10^8^	21.49%
3	70	M	IgG-κ	II	4	No	No	27.56%	0.88×10^8^	10.62%
4	55	F	Light chain κ	III	6	No	No	98.02%	1.57×10^8^	32.73%
5	54	M	IgD-λ	III	2	No	No	3.17%	4.55×10^8^	27.3%
6	58	M	Light chain κ	II	9	No	No	89.64%	1.38×10^8^	14.54%
7	66	M	Light chain λ	III	5	Yes	No	94.83%	1.16×10^8^	18.15%
8	54	F	IgG-λ	II	4	No	No	25.70%	1.5×10^8^	31.1%
9	58	F	IgD-λ	III	2	No	No	78.77%	1.22×10^8^	10.2%
10	62	F	IgG-λ	II	6	Yes	EM-B	33.52%	1.65×10^8^	11.1%
11	64	F	IgG-κ	II	5	No	No	78.40%	1.1×10^8^	8.8%

Extramedullary-extraosseous (EM-E): Hematogenous dissemination leads to soft tissue tumors at the anatomical site far from the bone. Extramedullary-bone related (EM-B): Breaks through the bone cortex and only invade the surrounding soft tissues.

**Table 2:** Baseline characteristics of patients.

Characteristics	Patients (N=11)
Age, median (range), years	58 (54–70)
Male gender, no. (%)	6 (54.5)
Monoclonal globulin, no. (%)	
IgG	4 (36.4)
IgD	2 (18.2)
IgM	0
Light chain	5 (45.5)
Non-secretory	0
ISS stage no. (%)	
I	1 (9.1)
II	6 (54.5)
III	4 (36.4)
EMD, no. (%)	2 (18.2)
BCMA expression, median% (range%)	66.23 (3.17–98.02)
Previous therapy lines, median (range)	4 (2–9)
Prior ASCT, no. (%)	2 (18.2)
β2-microglobulin, median (range), mg/L	2.7 (1.17–6.19)

### Long-term survival

**Figure 1:**
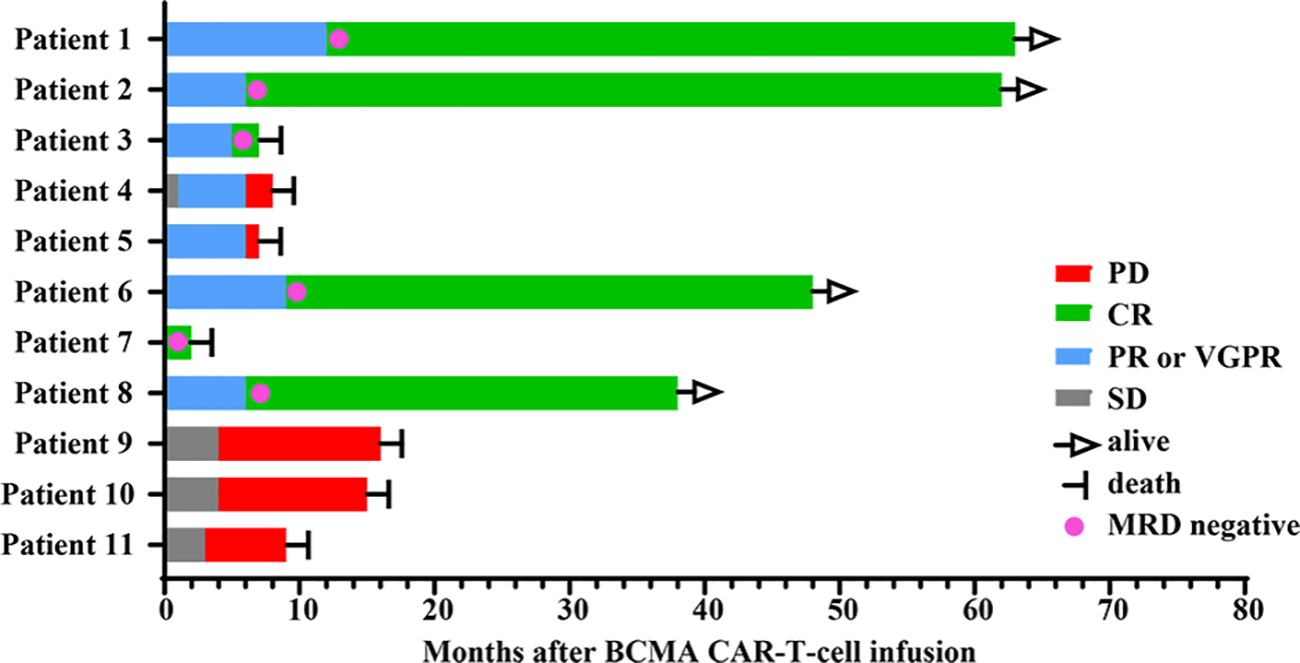
Treatment response and outcomes in 11 patients after BCMA CAR-T-cell infusion.

**Figure 2:**
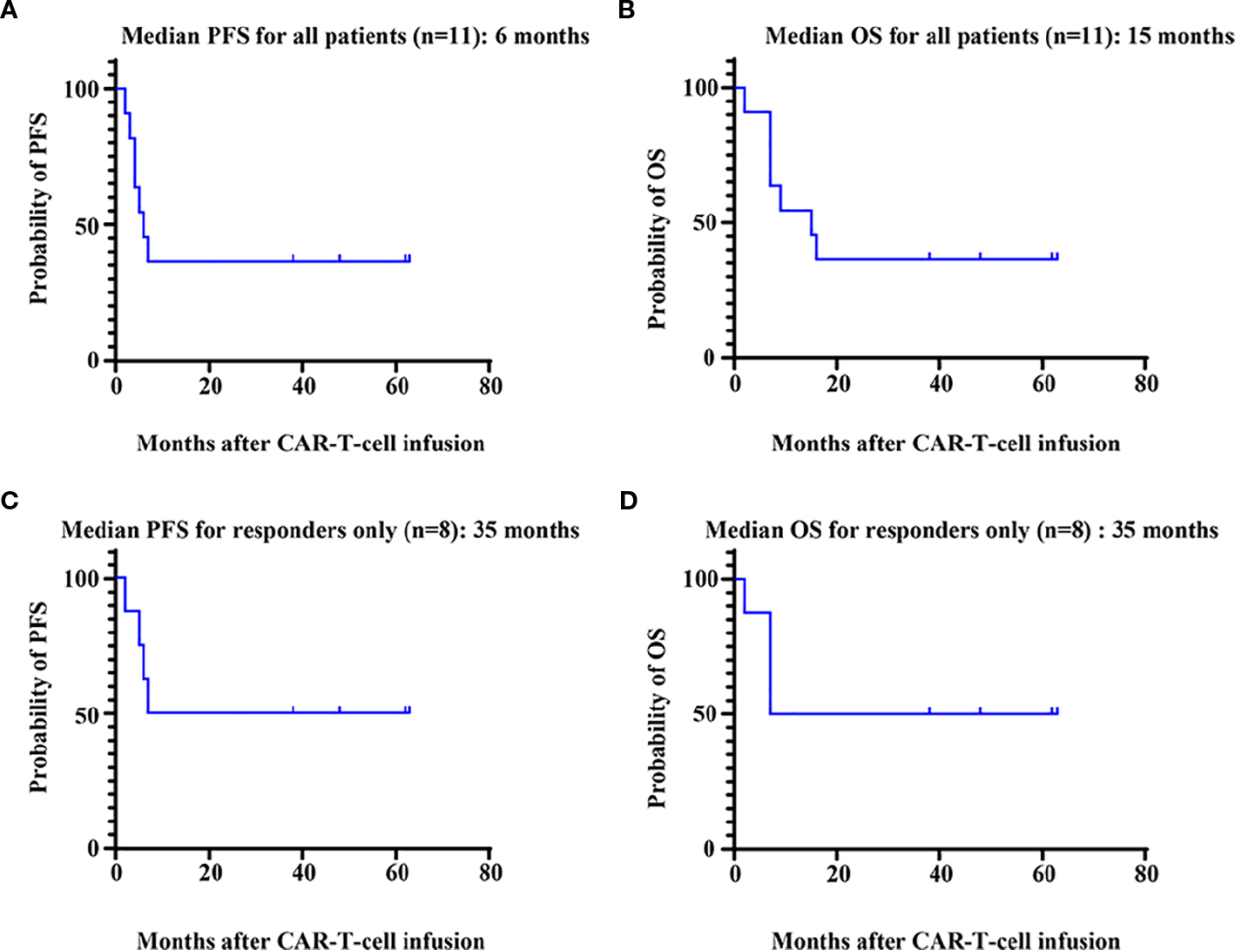
Survival analysis of patients. PFS **(A)** and OS **(B)** for all 11 patients who received BCMA CAR-T-cell therapy. PFS **(C)** and OS **(D)** for patients (n=8) who responded to BCMA CAR-T-cell therapy. PFS, progression-free survival; OS, overall survival.

Among the 11 patients, 8 (72.7%) exhibited treatment responses and were classified as responders, including 6 (54.5%) who achieved a complete response (CR) and 2 (18.2%) who achieved a partial response (PR) or a very good partial response (VGPR) (**Figure 1**). Three patients (27.3%) had stable disease (SD) and progressed within 3 months; thus, these patients were categorized as nonresponders to BCMA CAR-T-cell therapy. For all patients, the median PFS and OS were 6 months and 15 months, respectively (**Figures 2A, B**). For responders (n=8), the median follow-up duration was 23 months (range: 2–63 months). The long-term survival analysis revealed a median PFS of 35 months, which was comparable to the median OS (**Figures 2C, D**). Notably, as of the data cutoff date (February 28, 2025), all patients who achieved CR remained relapse-free. Among these, 4 patients were still alive, while 2 had died—one due to liver failure secondary to hepatitis B reactivation and the other due to cardiac arrest resulting from persistent infection. All patients with a PR or a VGPR eventually relapsed. Patient 4, who had achieved a PR, received a second BCMA CAR-T-cell infusion as salvage therapy after PD but exhibited no response and died one month later. Two patients with EMD exhibited distinct outcomes. Patient 2, who had pleural involvement (EM-E), achieved a CR following CAR-T-cell therapy and remains alive to date. In contrast, Patient 10, with tumor cell involvement in the soft tissue surrounding the left ribs (EM-B), did not respond to CAR-T-cell therapy and passed away shortly after treatment.

Among the 6 patients who achieved CR, all achieved MRD negativity at the 10^-4^ threshold. To investigate whether early achievement of MRD negativity is correlated with a better prognosis, we analyzed the relationship between the time to achieve MRD negativity and the long-term survival in patients who achieved CR. The results revealed that among patients who were still alive with persistent remission, the median time to reach MRD negativity was 9 months (range: 6–12 months). However, two patients who achieved MRD negativity earlier—one at 1 month and the other at 5 months—died shortly afterward.

Additionally, no patients developed secondary neoplasms during long-term follow-up.

### Adverse events

The most common adverse events (AEs) following the infusion of CAR-T cells were cytokine release syndrome (CRS) and hematologic toxicity (**Table 3**). All patients developed CRS, including 9 (81.8%) with grades 1–2 and 2 (18.2%) with grades 3–4 CRS. The two patients (Patient 7 and Patient 8) who developed grade 3–4 CRS experienced markedly elevated levels of multiple cytokines and were managed with corticosteroids and supportive care. Patient 8 received additional treatment with tocilizumab and recovered rapidly. Unfortunately, Patient 7 succumbed to cardiac arrest despite these interventions (**Supplementary Figure S1**).

**Table 3:** Adverse events during the treatment.

Adverse events	Grade 1–2	Grade 3–5
Cytokine release syndrome	9 (81.8%)	2 (18.2%)
Hematological adverse events		
Leukopenia	3 (27.3%)	8 (72.7%)
Anemia	6 (54.5%)	5 (45.5%)
Thrombocytopenia	2 (18.2%)	6 (54.5%)
Non-Haematological adverse events		
Fever	8 (72.7%)	1 (9%)
Nausea	3 (27.3%)	0
Vomiting	2 (18.2%)	0
Hypotension	1 (9%)	1 (9%)
Hypoxaemia	1 (9%)	1 (9%)
Aspartate aminotransferase increased	0	2 (18.2%)
Alanine aminotransferase increased	1 (9%)	1 (9%)
Infection	5 (45.5%)	3 (27.3%)
Neurologic events	0	0

**Figure 3:**
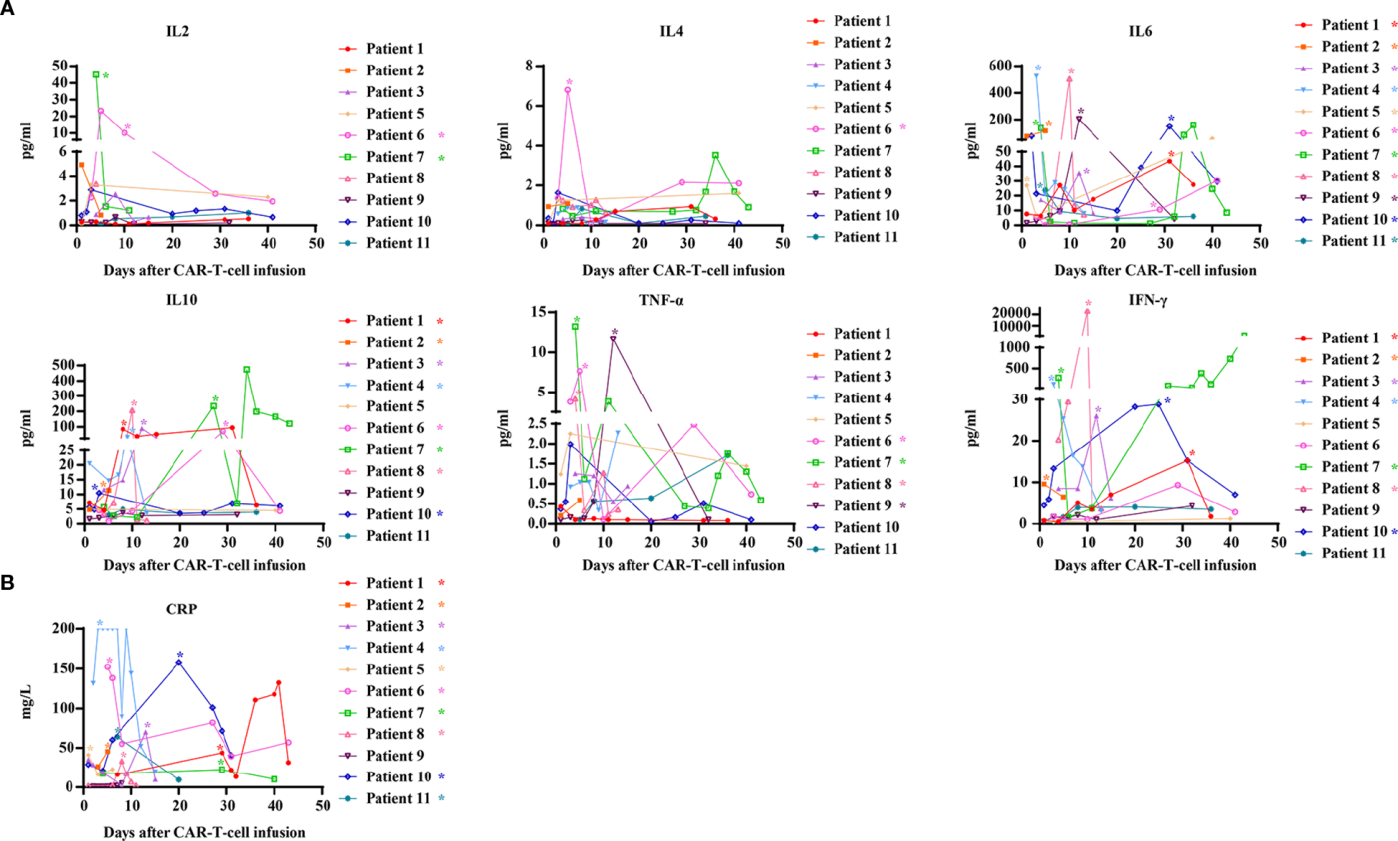
Kinetics of serum cytokines and CRP after CAR-T-cell infusion. **(A)** Kinetics of serum cytokines after CAR-T-cell infusion; **(B)** Kinetics of CRP after CAR-T-cell infusion. CRP, C-reactive protein. *Patients who exhibited elevated values within 31 days after infusion.

We also evaluated the serum levels of cytokines in all patients following CAR-T-cell infusion. Most cytokine levels peaked between 7 and 14 days after CAR-T-cell infusion but then gradually decreased. Within 31 days after CAR-T-cell infusion, elevated levels of interleukin-6 (IL-6), interleukin-10 (IL-10), tumor necrosis factor-α (TNF-α), and interferon-γ (IFN-γ) were observed in 11 (100%), 8 (72.7%), 4 (36.4%), and 7 (63.6%) patients, respectively. In contrast, increased levels of interleukin-2 (IL-2) and interleukin-4 (IL-4) were detected in only 2 patients and 1 patient, respectively (**Figure 3A**). C-reactive protein (CRP) levels were elevated in 10 patients (90.9%). CRP was moderately associated with CRS and gradually declined within 45 days (**Figure 3B**).

Hematological toxicity was observed in all patients during the first month after the infusion of CAR-T cells. In accordance with the National Cancer Institute Common Terminology Criteria for Adverse Events version 5.0 (CTCAE v5.0), grade 3–4 leukopenia, anemia, and thrombocytopenia were observed in 8 (72.7%), 5 (45.5%), and 6 (54.5%) patients, respectively (**Table 3**). With supportive treatment, hematological toxicity resolved to grade ≤2 within 31 days in 7 (63.6%) patients (**Supplementary Figure S2**). Five patients (Patients 3, 6, 7, 9, and 10) experienced prolonged cytopenia, which was defined as failure to resolve to grade ≤2 within one month. Four of these patients experienced resolution of cytopenia within 3 months after the infusion (**Supplementary Figure S3**). Although Patient 7 achieved a CR, this individual exhibited prolonged leukopenia and thrombocytopenia and succumbed to cardiac arrest secondary to persistent severe infection 1.5 months post-infusion. Notably, anemia and leukopenia were prevalent in most patients before or during pretreatment with fludarabine and cyclophosphamide, which suggests that hematological toxicity was not exclusively caused by CAR-T-cell therapy.

The most common nonhematological AEs were fever and infection. Fever was noted in 9 patients (81.8%), with only one case of grade 3 fever. Infection was observed in 8 (72.7%) patients, among whom 3 experienced grade 3 infections, primarily involving the lungs and gastrointestinal tract. Additionally, less common AEs, such as hallucinations and blurred vision, occurred in two patients. No patients developed neurologic events or immune effector cell-associated neurotoxicity syndrome (ICANS).

### The kinetics of CAR-T cells and B cells

**Figure 4:**
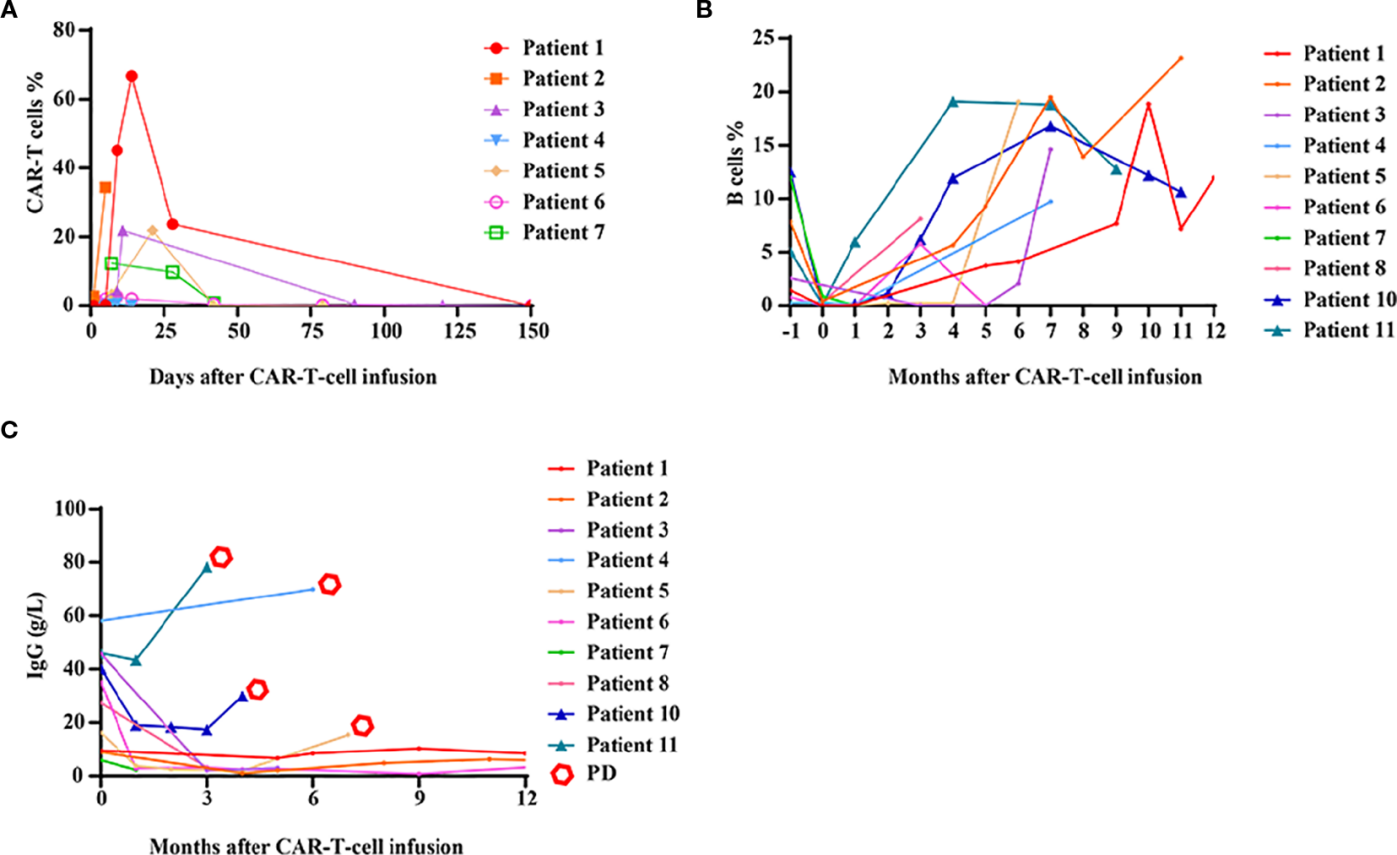
Kinetics of CAR-T cells, B cells and immunoglobulin G levels after CAR-T-cell infusion. **(A)** The expansion and persistence of BCMA CAR-T cells in patients who achieved a PR or better. **(B)** The numbers of B cells in peripheral blood. **(C)** The levels of immunoglobulin G (IgG) in peripheral blood.

In BCMA CAR-T responders, cell expansion peaked at 7–15 days, followed by a gradual decline within 1 month. CAR-T cells became undetectable in all patients after 1 month (**Figure 4A**). No significant correlation was observed between CAR-T-cell expansion/persistence and clinical outcomes.

Almost all patients exhibited B-cell aplasia within the first month, which gradually resolved over time. Compared with responders, patients who did not respond to CAR-T-cell therapy tended to exhibit earlier B-cell reconstitution, although this difference was not statistically significant (**Figure 4B**). Six months after CAR-T-cell infusion, most patients demonstrated evidence of B-cell reconstitution, and seven patients had B-cell counts within the normal range. Immunoglobulin G (IgG) recovery lagged behind B-cell recovery. Notably, in patients with durable remission, IgG levels remained persistently low. In contrast, for patients who experienced disease relapse or progression, IgG levels rapidly increased (**Figure 4C**).

## Discussion

In our single-center study, we present real-world long-term follow-up data of 11 patients with R/R MM treated with BCMA CAR-T-cell therapy. The patients were predominantly elderly, with a median age of 58 (range: 54–70) years, and were resistant to at least one IMiD or PI. The median PFS and OS for all patients in this study were 6 months and 15 months, respectively. Among responders, both the median PFS and OS reached 35 months. In contrast, previous studies have reported a median PFS ranging from 8.8–22.6 months and a median OS of 34.2–55.8 months, which highlights potential differences in survival outcomes that may warrant further investigation into underlying factors such as the differentiation of CAR-T cells, treatment methods, or disease stage (15–17). Notably, as of the data cutoff date, four patients remained alive with ongoing responses for more than 3 years, and the longest remission duration was 5.3 years.

MRD-negative status is a crucial determinant of response depth and is associated with durable remission (18). In our study, 6 patients achieved MRD negativity. Intriguingly, early attainment of an MRD-negative status was not necessarily correlated with favorable long-term outcomes. The median time for the 4 patients who were still alive with persistent remission to achieve MRD negativity was 9 months (range: 6–12 months). Conversely, two patients who achieved MRD negativity earlier both died shortly thereafter. Gradual onset of the best response resulted in a better prognosis, which is consistent with the findings of other studies (17, 19). Multiple factors are associated with response depth and duration, including the CAR-T-cell infusion dose, prior exposure to CAR-T-cell therapy, number of previous treatment lines, and the tumor microenvironment (TME) (20). Our experience indicated that the infusion dose (1–5×10^6^ cells/kg) was weakly correlated with remission depth and duration but exacerbated toxicity. BCMA CAR-T-cell reinfusion after relapse, as observed in patient 4, conferred limited benefits. Additionally, no significant association was found between the number of previous treatment lines and treatment efficacy.

The sustained persistence of CAR-T cells plays a pivotal role in determining long-term outcomes (21, 22). Melenhorst et al. (23) reported that two patients with CLL who had a sustained CR had detectable CAR-T cells for more than a decade, which represents the longest reported persistence of CAR-T cells to date. Yu et al. (16) reported that CAR-T cells remained detectable in most patients who achieved a CR during a median follow-up period of 45 months, whereas CAR-T cells exhibited poor persistence and expansion in patients with PD or relapse. However, in the present study, CAR-T cells were not detected in 5 of the 8 patients who achieved a PR or better by day 30. By 5 months post-infusion, CAR-T cells had become completely undetectable in all patients. To promote the expansion of CAR-T cells, we attempted to infuse recombinant human interleukin-2, but this intervention was ineffective. Interestingly, despite the undetectability of CAR-T cells, 4 patients remained alive with CR, which suggests that, to some extent, the persistence time of CAR-T cells may not be correlated with long-term outcomes. These findings align with those of the LEGEND-2 trial, which demonstrated that the long-term persistence of CAR-T cells did not necessarily result in durable remission (17). One possible explanation for the failure to detect CAR-T cells is that residual CAR-T cells were below the sensitivity threshold of FCM. Monitoring CAR-T cells using digital droplet PCR (ddPCR) rather than FCM may increase sensitivity, enabling the detection of CAR-T cells even at lower copy numbers (16). Previous studies have identified long-lived CAR-T cells as a low-frequency subpopulation characterized predominantly by a CD4/CD8 double-negative (DN) phenotype (17). These DN CAR-T cells exhibit limited aggressive expansion capacity and are hypothesized to originate from early memory-like or naive CAR-T cells, as memory and naive T cells are known to be associated with enhanced *in vivo* expansion of CAR-T cells (24, 25). In this context, another potential explanation for the undetectability of long-lived CAR-T cells in our study could be the lower proportion of early memory-like T cells and naive T cells among our CAR-T-cell products. However, importantly, this possibility was not formally evaluated in our study, and thus this hypothesis requires further investigation.

The expansion and persistence of CAR-T cells *in vivo* are influenced by several factors, including intrinsic and extrinsic factors. Intrinsic factors, such as costimulatory molecules, transmembrane domains and endodomains, primarily affect CAR structure (26). Fourth-generation CARs that incorporate cytokines (IL-7, IL-15, or IL-21) increase CAR-T-cell persistence, tumor targeting, and antitumor activity (27). Extrinsic factors such as the TME and anti-drug antibodies (ADAs) may impede the proliferation of CAR-T cells (28). Increasing numbers of studies have demonstrated that immune suppressor cells, including M2 macrophages, may establish a hostile TME and can thus mitigate CAR-T-cell activity and persistence (29). Although fully human-origin CARs lower the incidence of ADA more than their murine counterparts do, no established links have been reported between ADAs and CAR-T-cell kinetics (16).

The correlation between baseline BCMA intensity and CAR-T-cell therapeutic efficacy is debatable. While some studies have associated high BCMA expression in MM cells with effective CAR-T-cell therapy, others have reported no significant association (25, 30, 31). Our study revealed no significant association between baseline tumor BCMA expression and treatment efficacy, which indicates that BCMA expression does not limit the use of BCMA CAR-T cells. Preclinical studies suggest that EMD is a poor prognostic factor for MM in BCMA CAR-T-cell-treated patients (32, 33). In the current study, two patients with EMD exhibited distinct outcomes. Patient 2 remained disease-free without progression, while Patient 10 experienced PD within a short period and died shortly thereafter. Several potential factors may influence the prognosis of these two patients. For instance, Patient 10 had undergone more prior treatment lines compared to Patient 2 (6 versus 4). Notably, Patient 10 had also received an ASCT prior to CAR-T therapy—a factor that may have impaired the quality of the T cells, potentially compromising the functionality and persistence of the infused CAR-T cells (8). Furthermore, Patient 10 received a lower quantity of infused CAR-T cells, coupled with a lower transduction efficiency. Together, these factors may have directly reduced the therapeutic efficacy of the CAR-T cells, limiting its ability to control the extramedullary disease.

CAR-T-cell-related AEs are critical because of their common occurrence and potential lethality. In our study, although the incidence of CRS was 100%, most cases were grade 1–2 and resolved with supportive care. Patient 8 received tocilizumab for extremely high IL-6 and IFN-γ levels, which rapidly decreased after treatment. Hematological toxicity (leukopenia, anemia, thrombocytopenia) was common, likely because of bone marrow suppression from both CAR-T-cell infusion and lymphodepletion chemotherapy. Additionally, some patients had preexisting hematological abnormalities. In most patients, hematological toxicity resolved within 1 month. However, several patients experienced prolonged cytopenia, but most of these cases resolved within 3 months. An exception was Patient 7, who experienced persistent leukopenia for two months and ultimately died of cardiac arrest secondary to severe infection.

During long-term follow-up, none of the patients developed secondary neoplasia. Published data show that the 5-year incidence of secondary solid tumors after CAR-T-cell therapy ranges from 5.4–15.2% (17, 34). Recent studies by Harrison et al. (35) and Perica et al. (36) revealed that three MM patients developed T-cell lymphoma after they received cilta-cel CAR-T-cell therapy and that CAR transgene expression and integration were detected in monoclonal T cells. Whether this resulted from CAR vector integration, genomic alterations, viral infections, or other factors requires further investigation.

This study is subject to several limitations that should be considered when interpreting its findings. First, the small sample size (n=11) substantially constrains the statistical power, and thus it is challenging to draw definitive and robust conclusions about the efficacy and safety of the treatment. Small cohorts are more prone to random variation, which can limit the generalizability of the results to larger patient populations. Second, the use of different CAR-T-cell products across the included trials may introduce confounding variables that complicate interpretations of efficacy and safety outcomes. Specifically, in the present study, the single patient enrolled in the NCT03975907 trial might have inadvertently skewed the assessment of long-term efficacy, given the lack of a larger comparator group treated with the same product. Finally, the COVID-19 pandemic disrupted routine disease monitoring protocols. As a result, some patients underwent follow-up evaluations at local hospitals, which may have led to incomplete or inconsistent data collection.

## Conclusion

BCMA CAR-T-cell therapy is a safe and effective treatment for advanced R/R MM. Long-term follow-up revealed that BCMA CAR-T cells elicited a durable response in 50% of advanced R/R MM patients who responded.

## Data Availability

The original contributions presented in the study are included in the article/**Supplementary Material**. Further inquiries can be directed to the corresponding authors.

## Supplementary material

Supplementary Figure 1 Kinetics of serum cytokines and CRP after CAR-T-cell infusion in two patients who experienced grade 3–4 CRS. *Patients who exhibited elevated values within 31 days after infusion.

Supplementary Figure 2 Kinetics of leukocyte, hemoglobin, and platelet in all patients after CAR-T-cell infusion. The kinetics of WBC **(A)**, HGB **(B)**, and PLT **(C)** in all patients after CAR-T-cell infusion. WBC, leukocyte; HGB, hemoglobin; PLT, platelet. *Patients who failed to resolve to grade ≤2 within one month.

Supplementary Figure 3 Kinetics of leukocyte, hemoglobin, and platelet in patients with prolonged cytopenia. The kinetics of WBC **(A)**, HGB **(B)**, and PLT **(C)** in patients with prolonged cytopenia. WBC, leukocyte; HGB, hemoglobin; PLT, platelet.
