# Supplementary figures and images for: Long-term follow-up of patients with relapsed/refractory multiple myeloma after BCMA CAR-T-cell therapy
Source: Front Immunol. 2025 Sep 12;16:1650568. doi: 10.3389/fimmu.2025.1650568 (PMC12463850; doi:10.3389/fimmu.2025.1650568)

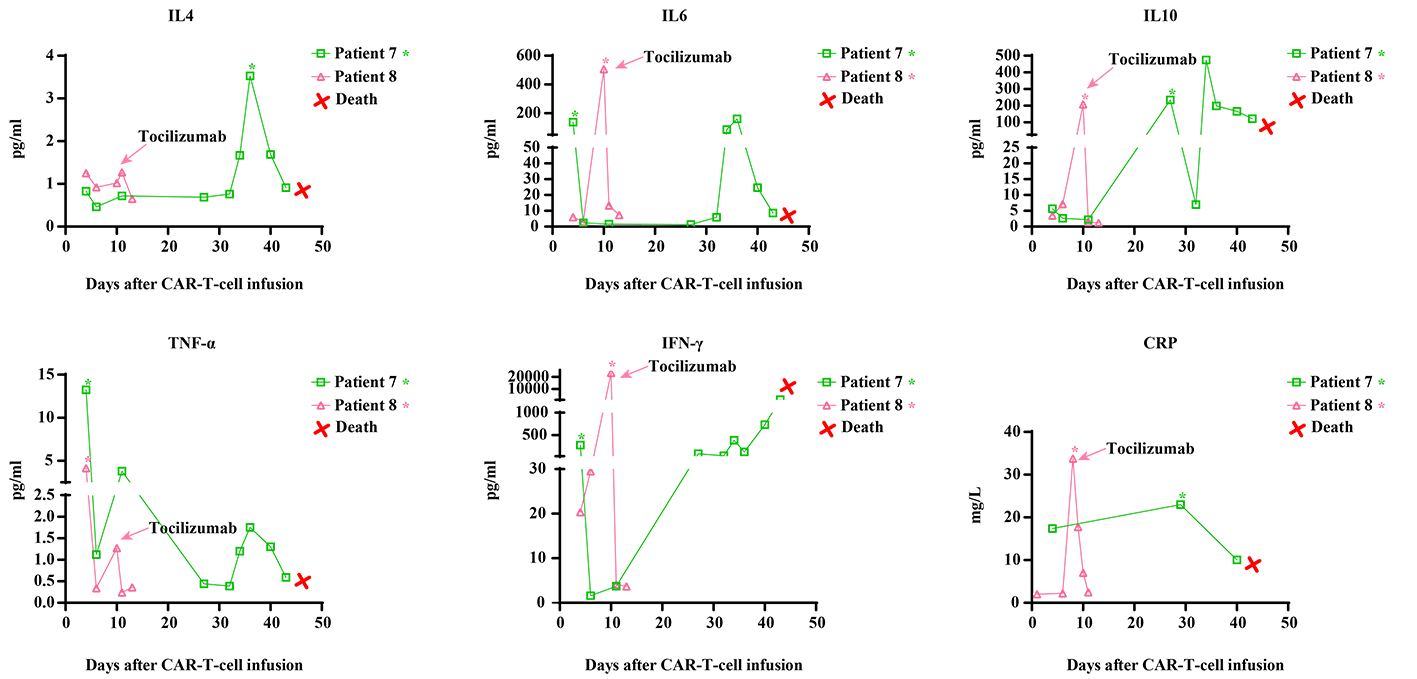

Supplement: Supplementary Figure 1 — Kinetics of serum cytokines and CRP after CAR-T-cell infusion in two patients who experienced grade 3–4 CRS. *Patients who exhibited elevated values within 31 days after infusion. [file Image1.tif]

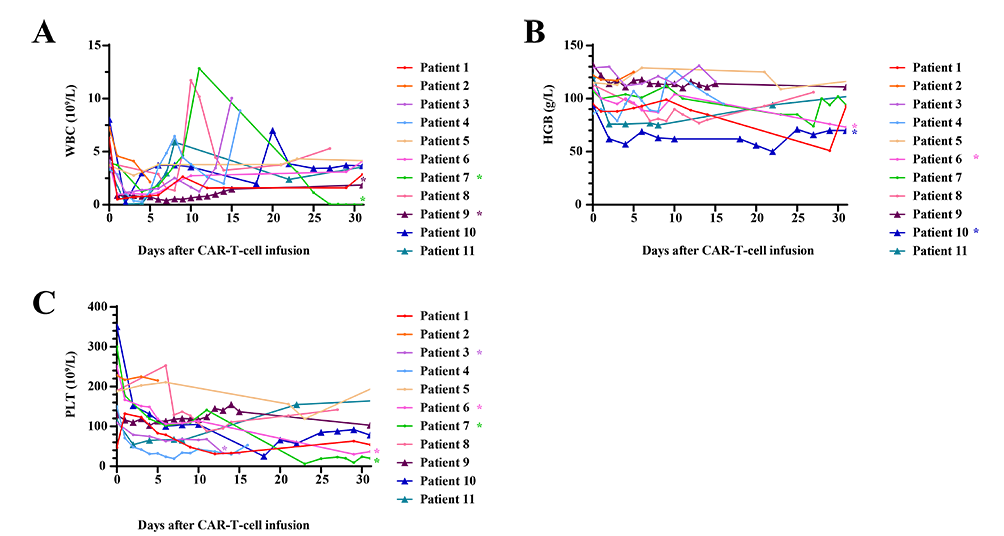

Supplement: Supplementary Figure 2 — Kinetics of leukocyte, hemoglobin, and platelet in all patients after CAR-T-cell infusion. The kinetics of WBC (A), HGB (B), and PLT (C) in all patients after CAR-T-cell infusion. WBC, leukocyte; HGB, hemoglobin; PLT, platelet. *Patients who failed to resolve to grade ≤2 within one month. [file Image2.tif]

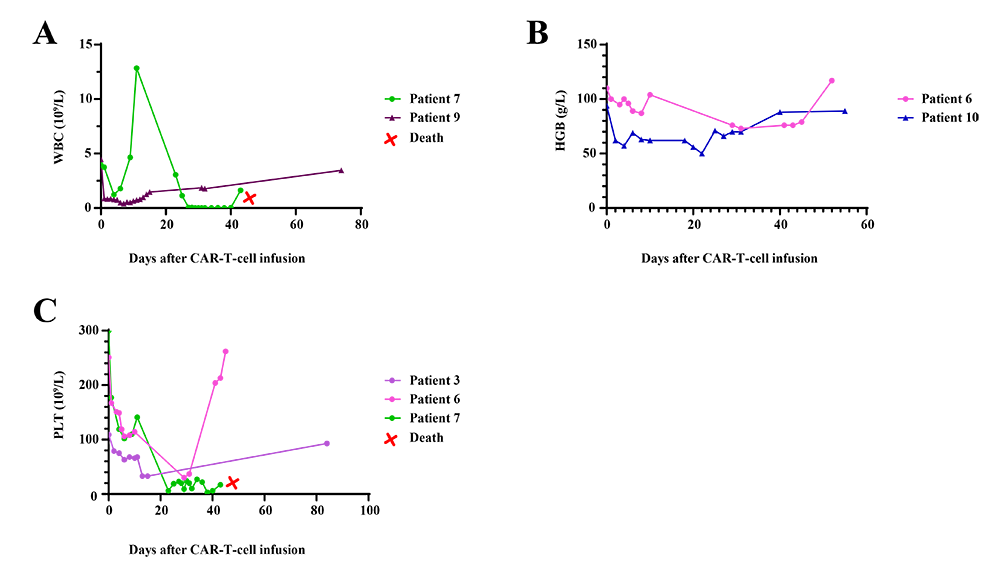

Supplement: Supplementary Figure 3 — Kinetics of leukocyte, hemoglobin, and platelet in patients with prolonged cytopenia. The kinetics of WBC (A), HGB (B), and PLT (C) in patients with prolonged cytopenia. WBC, leukocyte; HGB, hemoglobin; PLT, platelet. [file Image3.tif]
